# A R2R3-MYB Transcription Factor Regulates the Flavonol Biosynthetic Pathway in a Traditional Chinese Medicinal Plant, *Epimedium sagittatum*

**DOI:** 10.3389/fpls.2016.01089

**Published:** 2016-07-21

**Authors:** Wenjun Huang, A. B. M. Khaldun, Jianjun Chen, Chanjuan Zhang, Haiyan Lv, Ling Yuan, Ying Wang

**Affiliations:** ^1^Key Laboratory of Plant Germplasm Enhancement and Specialty Agriculture, Wuhan Botanical Garden – Chinese Academy of SciencesWuhan, China; ^2^Key Laboratory of Oil Crop Biology of the Ministry of Agriculture, Oil Crops Research Institute of the Chinese Academy of Agriculture SciencesWuhan, China; ^3^Department of Plant and Soil Sciences, University of Kentucky, LexingtonKY, USA

**Keywords:** *Epimedium*, flavonoid, flavonol, MYB, transcription factor

## Abstract

Flavonols as plant secondary metabolites with vital roles in plant development and defense against UV light, have been demonstrated to be the main bioactive components (BCs) in the genus *Epimedium* plants, several species of which are used as materials for *Herba Epimedii*, an important traditional Chinese medicine. The flavonol biosynthetic pathway genes had been already isolated from *Epimedium sagittatum*, but a *R2R3-MYB* transcription factor regulating the flavonol synthesis has not been functionally characterized so far in *Epimedium* plants. In this study, we isolated and characterized the *R2R3-MYB* transcription factor *EsMYBF1* involved in regulation of the flavonol biosynthetic pathway from *E. sagittatum*. Sequence analysis indicated that EsMYBF1 belongs to the subgroup 7 of R2R3-MYB family which contains the flavonol-specific MYB regulators identified to date. Transient reporter assay showed that *EsMYBF1* strongly activated the promoters of *EsF3H* (flavanone 3-hydroxylase) and *EsFLS* (flavonol synthase), but not the promoters of *EsDFRs* (dihydroflavonol 4-reductase) and *EsANS* (anthocyanidin synthase) in transiently transformed *Nicotiana benthamiana* leaves. Both yeast two-hybrid assay and transient reporter assay validated *EsMYBF1* to be independent of *EsTT8*, or *AtTT8 bHLH* regulators of the flavonoid pathway as cofactors. Ectopic expression of *EsMYBF1* in transgenic tobacco resulted in the increased flavonol content and the decreased anthocyanin content in flowers. Correspondingly, the structural genes involved in flavonol synthesis were upregulated in the *EsMYBF1* overexpression lines, including *NtCHS* (chalcone synthase), *NtCHI* (chalcone isomerase), *NtF3H* and *NtFLS*, whereas the late biosynthetic genes of the anthocyanin pathway (*NtDFR* and *NtANS*) were remarkably downregulated, compared to the controls. These results suggest that *EsMYBF1* is a flavonol-specific *R2R3-MYB* regulator, and involved in regulation of the biosynthesis of the flavonol-derived BCs in *E. sagittatum*. Thus, identification and functional characterization of *EsMYBF1* provide insight into understanding the biosynthesis and regulation of the flavonol-derived BCs in *Epimedium* plants, and also provide an effective tool gene for genetic manipulation to improve the flavonol synthesis.

## Introduction

Flavonoids are a large class of plant secondary metabolites, and play diverse roles in plant development and defense. They are derived from phenylalanine and divided into three major classes: anthocyanins, proanthocyanidins (PAs), and flavonols. For plant itself, the most prominent function of flavonols is to protect plant against UV light damage. While for human health, flavonols are shown to possess antiinflammatory, antioxidant, and antiproliferative capacities (Havsteen, 2002; Yao et al., 2004). Flavonoids, including flavonols attract a growing number of interests from public due to their significant benefits on human health.

The medicinal plants are generally known for the abundance of certain bioactive compounds, such as flavonoids, alkaloids, and terpenoids. For an example, *Herba Epimedii*, an important traditional Chinese medicine, is featured by icariin, a C8-prenylated flavonol glycoside (Ma et al., 2011). *Herba Epimedii* is prepared from the dried aerial parts of *Epimedium* species in the family Berberidaceae (Li et al., 2005). *Herba Epimedii*, traditionally used as a kidney tonic and antirheumatic medicinal herb for more than 2000 years, is also currently used for treating many other disease, including sexual dysfunction, osteoporosis, diabetics, cardiovascular disease, tumors, and immunoregulation (Ma et al., 2011; Li et al., 2015a; Jiang et al., 2016). Flavonoids, especially C8-prenylated flavonol glycosides have been demonstrated to be the main bioactive components (BCs) in *Epimedium* plants, such as icariin which is one of the most studied BCs with extensive therapeutic capacities including osteoprotective effect, cardiovascular protective effect, anti-cancer effect, and anti-inflammation effect (Ma et al., 2011; Jiang et al., 2015; Li et al., 2015a). Although the main flavonol-derived BCs have been phytochemically and pharmacologically well-characterized, and their molecular biosynthetic pathways have been clarified recently (Zeng et al., 2013; Huang et al., 2015), but the studies of regulation of the synthesis of these flavonols in *Epimedium* plants are scarcely reported.

As we know, flavonol biosynthesis is one of the branches of the flavonoid biosynthetic pathway. The flavonoid biosynthetic pathway has been extensively studied in several model plant species, including *Arabidopsis*, petunia, maize, and grape (Winkel-Shirley, 2001; Koes et al., 2005). Recently, in *Epimedium* plants, the flavonoid biosynthetic pathway has also been elucidated, and most of structural genes of this pathway has been isolated (Zeng et al., 2013; Huang et al., 2015). Moreover, it is well-established that the flavonoid biosynthetic pathway is predominantly regulated by *MYB*, *bHLH*, and *WD40* regulatory genes at the transcriptional level (Ramsay and Glover, 2005; Hichri et al., 2011). An increasing number of *MYB* TFs regulating the flavonoid biosynthetic pathway have been identified from many plant species, especially *Arabidopsis*, maize, petunia, snapdragon, and grape with abundant functionally characterized *MYB* regulators (Liu et al., 2015). Each specific branch of the flavonoid pathway in both *Arabidopsis* and grape is generally regulated by a different MYB regulator. There are *AtPAP1* and *AtPAP2* from *Arabidopsis* (Borevitz et al., 2000), *VvMYBA1* and *VvMYBA2* (Kobayashi et al., 2004; Walker et al., 2007) from grape identified to regulate anthocyanin biosynthetic branch, *Arabidopsis AtTT2* (Nesi et al., 2001) and grape *VvMYBPA1*, *VvMYBPA2* (Bogs et al., 2007; Terrier et al., 2009) identified to regulate proanthocyanidin (PA) biosynthetic branch, while *AtMYB12/AtMYB11/AtMYB111* (Mehrtens et al., 2005; Stracke et al., 2007) and *VvMYBF1* (Czemmel et al., 2009) identified to regulate flavonol biosynthetic branch. Corresponding, in *Epimedium* plants, two *R2R3-MYB* regulators, *EsMYBA1* and *EsAN2* were recently characterized to regulate anthocyanin biosynthetic pathway in a different tissue-specific manner (Huang et al., 2013, 2016). The correlation of gene-to-metabolite during leaf developmental stages of *Epimedium sagittatum* has been systematically analyzed in the survey (Huang et al., 2015). A *R2R3-MYB* TF out of a dozen of *MYB* TFs studied was found to basically correlate with the accumulation patterns of the main four BCs (epimedin A, B, C and icariin) and co-express with several the flavonol biosynthetic pathway genes. The primary results suggest that this *MYB* gene is probably involved in regulating the flavonol pathway (Huang et al., 2015). Due to the high level of sequence similarity with grape *VvMYBF1*, this *MYB* gene from *E. sagittatum* was designated as *EsMYBF1* gene. However, the functional characterization of *EsMYBF1* gene is still far from being clarified.

In regulation of the anthocyanin and PA biosynthetic pathways, MYB TFs generally interact with bHLH TFs and WD40 proteins to form a MYB-bHLH-WD40 (MBW) complex (Xu et al., 2015). For example in *Arabidopsis*, *AtPAP1*/*AtPAP2-AtGL3*/*AtEGL3-AtTTG1* complex regulates anthocyanin synthesis (Gonzalez et al., 2008). However, in regulation of the flavonol biosynthetic pathway, *R2R3-MYB* regulators identified to date are found to be independent of *bHLH* cofactor. Three R2R3-MYB members (*AtMYB12/11/111*) of *Arabidopsis* and *VvMYBF1* of grape function as a activator of flavonol synthesis without binding a *bHLH* partner (Mehrtens et al., 2005; Stracke et al., 2007; Czemmel et al., 2009), probably because they don’t have the conserved [DE]Lx_2_[RK]x_3_Lx_6_Lx_3_R region described previously by Zimmermann et al. (2004) for interacting with bHLH regulators, which is generally required for the *MYB* regulators of both anthocyanin and PA biosynthesis. Moreover, these flavonol-specific *MYB* regulators can activate the promoters of target genes involved in flavonol biosynthesis, including *CHS*, *CHI*, *F3H*, and *FLS* (Mehrtens et al., 2005; Czemmel et al., 2009). Therefore, it is worthy to investigate whether or not the *EsMYBF1* gene is independent of *bHLH* cofactor and specifically regulates the flavonol biosynthetic pathway genes.

Majority of the flavonoid biosynthetic pathway genes in *Epimedium* have been isolated, but two *MYB* regulators of the flavonoid pathway reported to date are just confined to the anthocyanin synthesis, including *EsMYBA1* and *EsAN2* regulators (Huang et al., 2013, 2016). Material supply of *Herba Epimedii* mostly depends on wild resource of *Epimedium* plants, and certain species have been threatened with risk of extinction due to over-harvesting (Li et al., 2005). The flavonol-derived BCs accumulate lowly in *Epimedium* plants, and the overexpression of a flavonol-specific *MYB* regulator in transgenic plants is a powerful alternative way to improve the biosynthesis and accumulation of the desired flavonols. However, the putative *EsMYBF1* TF regulating flavonol biosynthesis has not been functionally characterized so far in *Epimedium* or any other medicinal plants until now. Herein, the putative flavonol-specific *EsMYBF1* regulator would be functionally characterized in-depthly. The results indicate that *EsMYBF1* is mainly expressed in leaves, and correlates with the accumulation of the main BCs during leaf development. *EsMYBF1* strongly activates the promoters of *EsF3H* and *EsFLS* genes involved in the flavonol biosynthesis rather than the promoters of *EsDFRs* and *EsANS* leading to the anthocyanin synthesis. Overexpression of *EsMYBF1* in tobacco results in the enhanced flavonol content and the reduced anthocyanin content, through upregulating the flavonol pathway genes and downregulating the late anthocyanin biosynthetic genes. The functional characterization of *EsMYBF1* regulator of flavonol synthesis makes a further step forward in understanding the regulatory mechanism of the flavonol-derived BCs biosynthesis in *Epimedium* plants, and also provides an effective tool gene for genetic manipulation to create new cultivars.

## Results

### Sequence Analysis and Expression Pattern of *EsMYBF1* Gene

**FIGURE 1:**
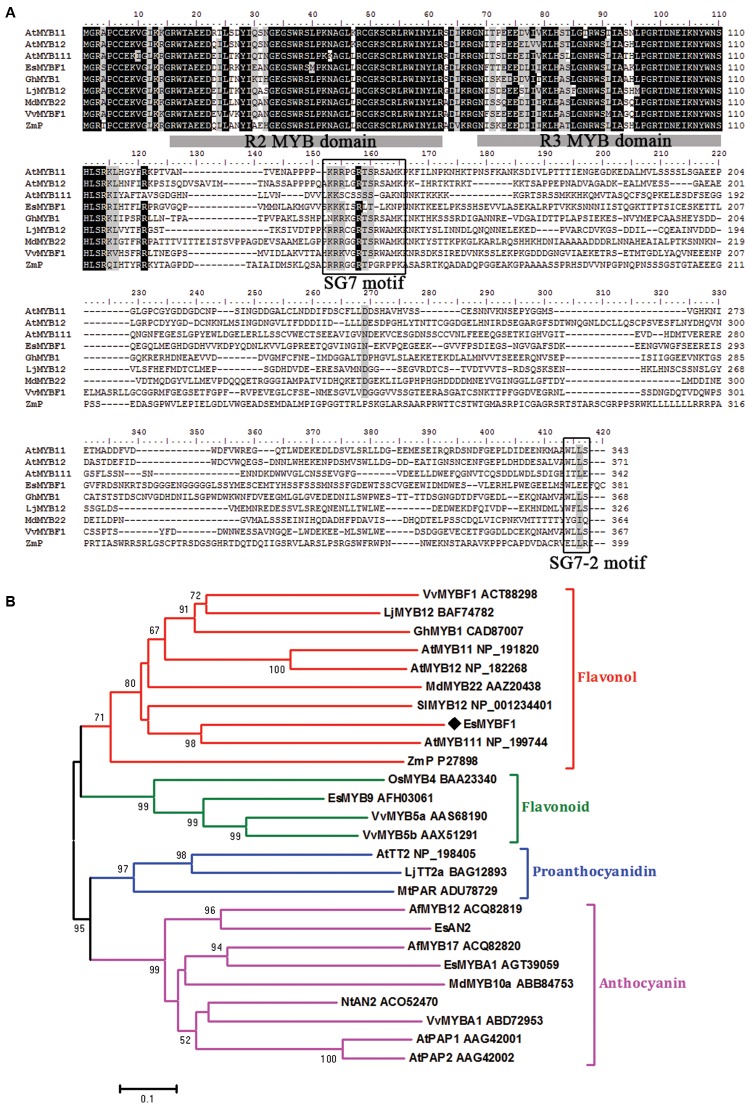
**Multiple alignment and phylogenetic analysis of EsMYBF1 and other plant R2R3-MYB proteins known to regulate the flavonoid biosynthetic pathway.** **(A)** Alignment of deduced amino acid sequences of EsMYBF1 and selected R2R3-MYB regulators of flavonol biosynthesis from other plant species. Identical amino acid residues are shaded in black, and similar in gray. The R2 and R3 MYB domains shown refer to two repeats of the MYB DNA-binding domain of MYB proteins. Two conserved motifs, SG7 and SG7-2 motifs are boxed. **(B)** Phylogenetic analysis of EsMYBF1 and selected R2R3-MYB proteins from other plant species. The phylogenetic tree is constructed using the neighbor-joining method by MEGA 5 software. The scale bar represents 0.1 substitution per site, and the numbers next to nodes are bootstrap values from 1,000 replicates. Bootstrap values less than 50 are not shown. EsMYBF1 is indicated as a diamond. Functions of most of the R2R3-MYB proteins are indicated. All R2R3-MYB protein sequences are retrieved from GenBank database, and their accession number are embedded in the diagram. Plant species are as follows: *Aquilegia formosa*, *Arabidopsis thaliana*, *Epimedium sagittatum*, *Gerbera hybrid*, *Lotus japonicus*, *Malus x domestica*, *Medicago truncatula*, *Nicotiana tabacum*, *Oryza sativa*, *Solanum lycopersicum*, *Vitis vinifera*, *Zea mays*.

The full-length cDNA clone of *EsMYBF1* was successfully obtained from *E. sagittatum* leaves using RACE (Rapid Amplification of cDNA Ends) method. *EsMYBF1* was predicted to have an entire ORF of 1146 bp length, encoding a R2R3-MYB protein of 381 amino acid residues in length. Sequence analysis revealed that EsMYBF1 contains the R2 and R3 MYB DNA-binding domains in its N-terminus (**Figure 1A**). The redefined SG7 motif ([K/R][R/x][R/K]xGRT[S/x][R/G]xx[M/x]K) characteristic of flavonol regulators of *Arabidopsis* and *Vitis vinifera* (Czemmel et al., 2009) was also found to be present in the C-terminus of VvMYBF1 (**Figure 1A**). Besides, EsMYBF1 also contains a similar counterpart (WLEE) of the SG7-2 motif ([W/x][L/x]LS) identified previously (Czemmel et al., 2009) in the C-terminus. Additionally, it is worthy to notice that the R2R3 repeat region of EsMYBF1 does not contain the motif [D/E]Lx_2_[R/K]x_3_Lx_6_Lx_3_R for interaction with bHLH partners (Zimmermann et al., 2004). Sequence similarity between MYB proteins is generally confined to the R2 and R3 repeats. EsMYBF1 showed high identity with other MYB regulators within the R2 and R3 MYB domains, such as 87% identity with apple MdMYB22 and 82% identity with *Arabidopsis* AtMYB12. However, within the overall protein sequence, EsMYBF1 showed only 44% identity to grape VvMYBF1, and 40% identity to *Arabidopsis* AtMYB111. Phylogenetic analysis indicated that EsMYBF1 was clustered with other flavonol MYB regulators together into the flavonol clade, and was closely related to *Arabidopsis* AtMYB111 regulator of flavonol synthesis (**Figure 1B**).

**FIGURE 2:**
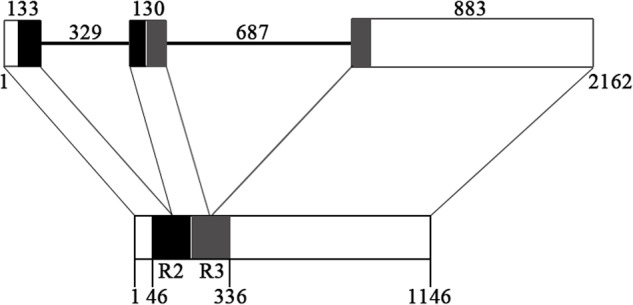
**Genomic structure of *EsMYBF1* gene.** The R2 and R3 MYB DNA-binding domains are shown as black and gray blocks, respectively. The exons are shown as blocks and the introns as lines. The diagram top represents the genomic sequence, and the numbers located above show the length of exons and introns. The bottom of diagram means the cDNA sequence, and the numbers located below show the distance from the start codon of *EsMYBF1* gene.

**FIGURE 3:**
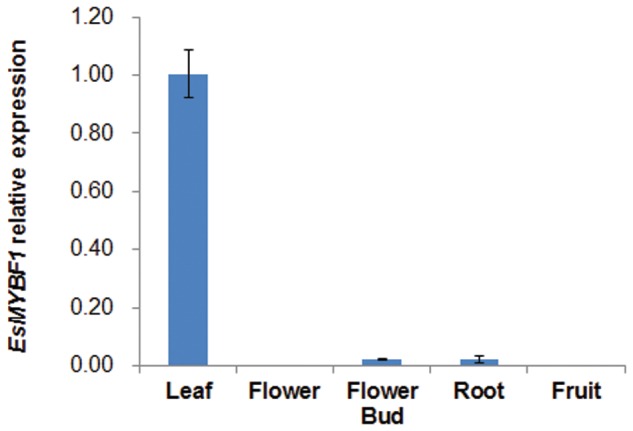
**Expression pattern of *EsMYBF1* in various tissues of *E. sagittatum*.** Transcript level of *EsMYBF1* is analyzed by quantitative RT-PCR assay. The comparative Ct method is used to determine the relative expression level. The column represents average value with SD bar from three technical replicates.

The genomic sequence of *EsMYBF1* was also isolated from genomic DNA of *E. sagittatum*. Alignment between the genomic and cDNA sequences showed that *EsMYBF1* consisted of three exons and two introns. These two introns were located into the R2 and R3 MYB domain, respectively (**Figure 2**). Moreover, the intron insertion places are highly conserved (data not shown). Additionally, to analyze the expression pattern of *EsMYBF1* in various tissues of *Epimedium*, qPCR assay was carried out. The results showed that *EsMYBF1* is mainly expressed in leaves, and very lowly expressed in other tissues (**Figure 3**).

### EsMYBF1 Is Independent of EsTT8 and AtTT8 bHLH Cofactors

**FIGURE 4:**
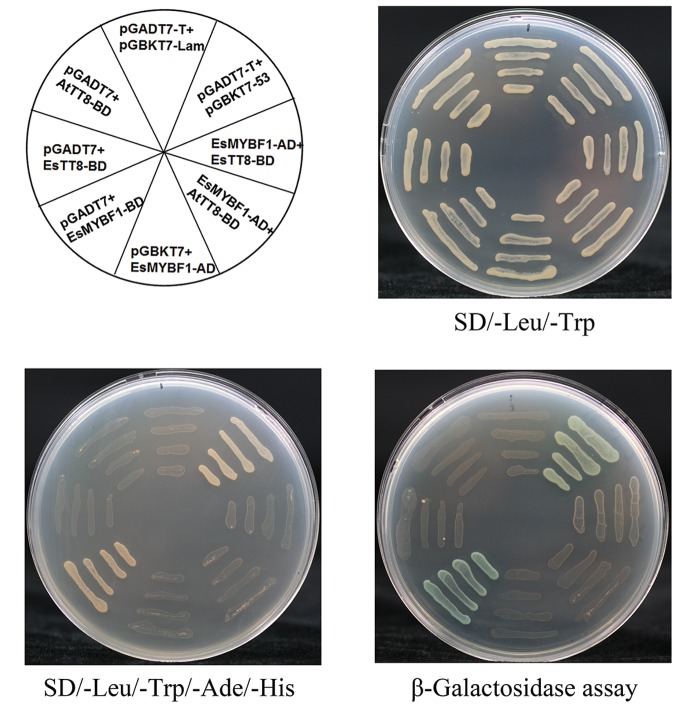
**Yeast two-hybrid assay of EsMYBF1 with two bHLH regulators of flavonoid pathway.** Two bHLH regulators involved in regulation of flavonoid biosynthetic pathway, EsTT8 from *E. sagittatum* and AtTT8 from *A. thaliana* are used for yeast two-hybrid assay. Transformed yeast cells containing pGBKT7-53+pGADT7-T, pGBKT7-Lam+pGADT7-T constructs are used as a positive and a negative control, respectively.

It is previously reported that the flavonol-specific *MYB* regulators are independent of *bHLH* cofactors. In order to validate whether or not *EsMYBF1* also has this characterization, Y2H assay of EsMYBF1 with AtTT8 or EsTT8 bHLH regulators was performed. The results showed that a total of eight combinations was able to grow on the double SD medium, but only two combinations, including the positive control (pGBKT7-53 + pGADT7-T) and the EsMYBF1-BD + pGADT7 constructs, grew on the quadruple SD medium (**Figure 4**). These results suggest that EsMYBF1 has a strong autoactivation activity, and does not interact with AtTT8 or EsTT8 bHLH regulators. This conclusion was further supported by the formation of blue colonies in the corresponding yeast cells through β-galactosidase assay (**Figure 4**). Finally, *EsMYBF1* is demonstrated to be independent of *bHLH* cofactor.

### *EsMYBF1* Activates Promoters of the Flavonol Pathway Genes

**FIGURE 5:**
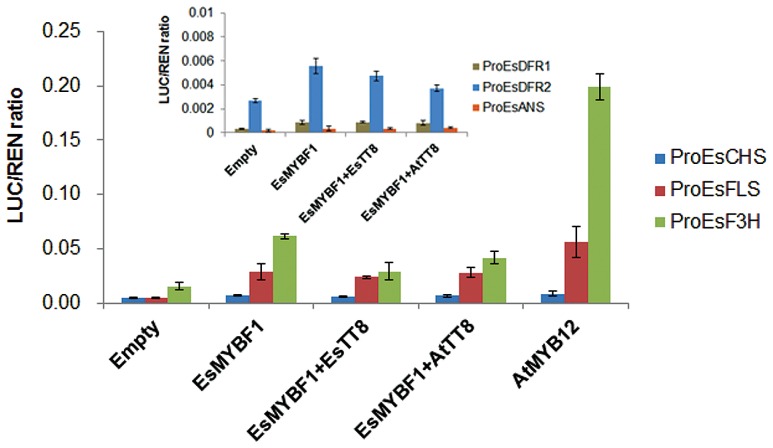
**Transcription activity assay of *EsMYBF1* against promoters of flavonoid-related genes with and without *bHLH* regulators in transiently transfected *Nicotiana benthamiana* leaves.** Six promoters of flavonoid biosynthetic genes from *E. sagittatum* are used for dual luciferase assay, including ProEsCHS, ProEsF3H, ProEsFLS, ProEsDFR1, ProEsDFR2, and ProEsANS. Two bHLH transcription factors involved in regulation of the flavonoid biosynthetic pathway, *EsTT8* from *E. sagittatum* and *AtTT8* from *A. thaliana* are also used as cofactors. Transformed leaves carrying only the promoter-LUC reporter construct without the transcription factors-containing effectors are used as controls. The columns represent average values with SD bar from four biological replicates for each treatment.

To identify the target genes of the flavonoid pathway by *EsMYBF1* TF, the dual luciferase assay system in transiently transformed *Nicotiana benthamiana* leaves was carried out. *EsCHS* and *EsF3H* as general flavonoid pathway genes are involved in the synthesis of flavonols, anthocyanins and PAs. Meanwhile, *EsDFRs* and *EsANS* are required for anthocyanin synthesis, whereas *EsFLS* as the flavonol specific branch point gene is specifically required for flavonol synthesis. Therefore, the promoters of these structural genes were chosen as potential targets of *EsMYBF1* transcription activation. The results showed that *EsMYBF1* strongly activated the promoters of both *EsF3H* and *EsFLS*, and slightly activated the promoter of *EsCHS* closely twofold, compared to the corresponding controls which only consisted of promoters without *MYB* TFs. Similarly, *AtMYB12* regulator of flavonol pathway as a positive control, also strongly activated the promoters of *EsF3H* and *EsFLS*, and slightly activated the promoter of *EsCHS* approximately twofold (**Figure 5**). However, the addition of *EsTT8* or *AtTT8*, a *bHLH* factor generally required as a cofactor for *MYB* transcription activator of flavonoid pathway, decreased the activation potential of *EsMYBF1* on the *EsF3H* promoter, or approximately equalized the activation of *EsMYBF1* on the promoters of both *EsFLS* and *EsCHS*, compared with *EsMYBF1* alone (**Figure 5**). In addition, the relative values of transcription activation of *EsMYBF1* alone or combined with *EsTT8* or *AtTT8 bHLH* regulators on the promoters of *EsDFR1*, *EsDFR2* and *EsANS* were approximately equal to that of the empty controls (promoter without *MYB* TF), and all these relative ratios were very close to zero (**Figure 5**), suggesting that *EsMYBF1* cannot activate the promoters of *EsDFRs* and *EsANS*. In summary, these results suggest that *EsMYBF1* is a specific regulator of flavonol synthesis, potentially regulating the *EsF3H* and *EsFLS* genes, and is independent of *EsTT8* or *AtTT8 bHLH* cofactors.

### Overexpression of *EsMYBF1* in Tobacco Affects the Accumulation of Flavonoids

**FIGURE 6:**
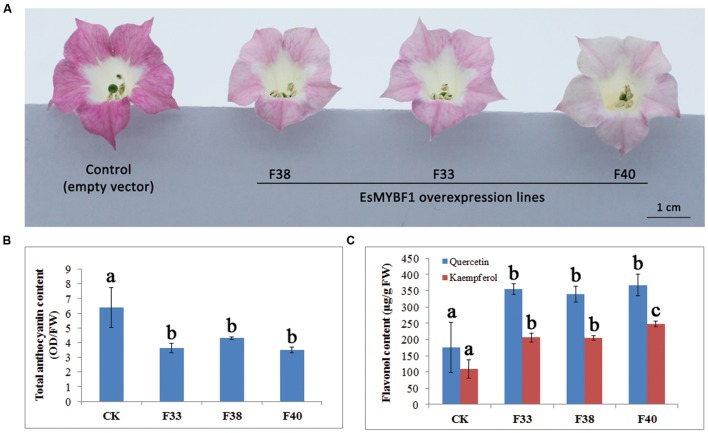
**Phenotype observation and flavonoid content measurement in transgenic tobacco overexpressing *EsMYBF1* gene.** Three independent transgenic tobacco lines carrying *EsMYBF1* (F33, F38, F40) and the transgenic tobacco carrying the empty vector as control plants are used. Phenotypic changes observation **(A)**, and total anthocyanin content measurement **(B)** and flavonol content measurement **(C)** are performed. Each column represents mean values with SD bar from five biological replicates for each sample. The significant difference is calculated by Duncan statistical analysis at the level of *P* ≤ 0.05.

To characterize the function of *EsMYBF1*, overexpression of *EsMYBF1* in tobacco was carried out. The results showed that flower color of transgenic tobacco carrying *EsMYBF1* turned from rosy red to light pink (**Figure 6A**). Among the three overexpression lines (F33, F38, and F40), flower color change of F40 lines was most strong. Corresponding to the phenotypic changes, flavonoid contents, including total anthocyanins and flavonols in transgenic tobacco flowers were measured. The results indicated that total anthocyanin content was significantly decreased in all three overexpression lines, compared to the controls (**Figure 6B**). However, flavonol content (kaempferol and quercetin) was significantly increased by twofold in all three overexpression lines, compared to the controls (**Figure 6C**). These results suggest ectopic expression of *EsMYBF1* in tobacco directed the metabolic flux from anthocyanin pathway to flavonol pathway.

### Overexpression of *EsMYBF1* in Tobacco Affects the Expression Levels of Flavonoid Pathway Genes

**FIGURE 7:**
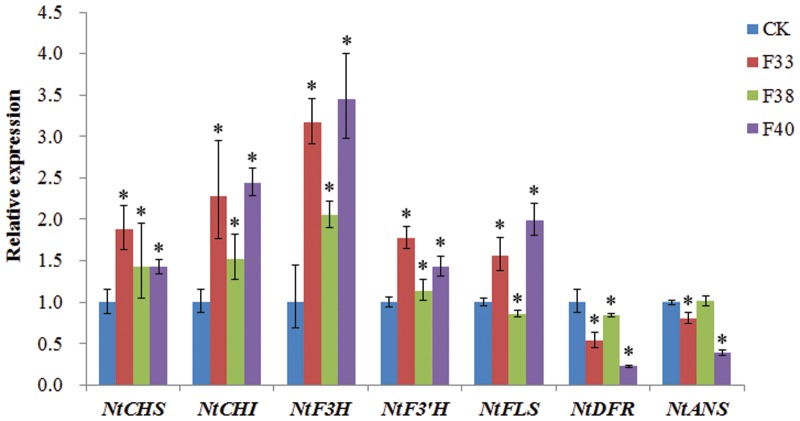
**Quantitative PCR assay of the flavonoid pathway genes in transgenic tobacco carrying *EsMYBF1* gene.** Transcript levels of seven structural genes of flavonoid biosynthetic pathway are determined by quantitative PCR assay, including *NtCHS*, *NtCHI*, *NtF3H*, *NtF3′H*, *NtFLS*, *NtDFR*, and *NtANS*. Tobacco *Tub1* gene is used as an internal control, and comparative Ct method is used to determine the relative expression level. The columns represent average value with SD bar from three technical replicates. Three *EsMYBF1* overexpressing tobacco lines (F33, F38, F40) and transgenic tobacco plants carrying the empty vector (CK) are used for qPCR assay. The significant difference of the three overexpression lines against the control line was analyzed using LSD test at the level of *P* ≤ 0.05. The ^∗^ symbol represents the significant difference at the level of *P* ≤ 0.05.

The effect of *EsMYBF1* overexpression on the expression levels of flavonoid pathway genes in tobacco flowers was analyzed by qPCR assay. The presence of *EsMYBF1* in transgenic tobacco was firstly confirmed by RT-PCR assay (data not shown). The qPCR results showed that five structural genes, including *NtCHS*, *NtCHI*, *NtF3H*, *NtF3′H* and *NtFLS*, were remarkably upregulated in two overexpression lines (F33 and F40), but *NtDFR* and *NtANS* was remarkably downregulated in two overexpression lines (F33 and F40), especially in F40 line, compared to the controls (**Figure 7**). Moreover, *NtF3H* was most highly increased, followed by *NtCHI* and *NtFLS*, particularly in F33 and F40 lines. It is notable that in F38 lines the change extent of gene expression was obviously lower than that in other two overexpression lines (**Figure 7**).

## Discussion

The functional similarity of *R2R3-MYB* TFs can be predicted by their structural similarity. The isolation of *Arabidopsis AtMYB12* was just derived from sequence similarity analysis with maize P regulator (Mehrtens et al., 2005), and *VvMYBF1* isolation was from *AtMYB12* homolog (Czemmel et al., 2009). In this study, the putative *EsMYBF1* regulator of flavonol pathway was also derived from these two *AtMYB12* and *VvMYBF1* homologs. EsMYBF1 shares the highly conserved R2 and R3 MYB domains in the N-terminus, and the redefined SG7 and SG7-2 motifs (Czemmel et al., 2009) in the diverse C-terminus with other flavonol-specific MYB regulators (**Figure 1A**). Additionally, the R2 and R3 domains of EsMYBF1 does not contain the conserved bHLH interaction motif identified previously (Zimmermann et al., 2004), suggesting that EsMYBF1 is bHLH cofactor independent. This cofactor independency is generally restricted to the MYB factors of the flavonol clade (**Figure 1B**). In consideration of the high structural similarity of EsMYBF1 and members of the MYB subgroup 7, EsMYBF1 is predicted to be a putative flavonol regulator of MYB subgroup 7.

As we know, the expression of the *MYB* regulator of flavonol pathway correlates well with the flavonol synthesis and accumulation, such as *Arabidopsis AtMYB12* and grapevine *VvMYBF1* (Mehrtens et al., 2005; Czemmel et al., 2009). It was previously reported that the expression pattern of *EsMYBF1* basically correlates with the accumulation pattern of the four flavonol-derived BCs during leaf development of *Epimedium* (Huang et al., 2015). Moreover, our results indicated that *EsMYBF1* is most abundantly expressed in leaves (**Figure 3**), where the main flavonol-derived BCs accumulate largely. These results suggest that *EsMYBF1* is involved in the developmental regulation of the flavonol-derived BCs in *Epimedium* leaves.

Both *AtMYB12* and *VvMYBF1* were previously reported to activate the flavonol pathway genes without the need of a *bHLH* cofactor (Mehrtens et al., 2005; Czemmel et al., 2009), probably because they do not contain the bHLH interaction motif in the R2R3 MYB domain. Similarly, the absence of the bHLH interaction motif in EsMYBF1 protein also contributed to that EsMYBF1 is independent of bHLH cofactors. It was demonstrated firstly that EsMYBF1 does not interact with the two bHLH regulators of flavonoid pathway in yeast two-hybrid assay, including EsTT8 from *E. sagittatum* and AtTT8 from *Arabidopsis* (**Figure 4**). Sequentially, the inclusion of *EsTT8* or *AtTT8 bHLH* regulator was not required for transcription activation by *EsMYBF1* in the transient reporter assay, and the presence of the *bHLH* actually slightly diminished activation (**Figure 5**). Although the promoter of *EsCHS* was slightly activated by *EsMYBF1*, the promoters of *EsF3H* and *EsFLS* was strongly activated by *EsMYBF1* (**Figure 5**), suggesting that *EsMYBF1* is able to control the entire pathway leading to flavonol synthesis. Similar results were previously reported for the control of flavonol synthesis by *AtMYB12/11/111* of *Arabidopsis* and *VvMYBF1* of grapevine (Stracke et al., 2007; Czemmel et al., 2009). In addition, the CHS enzyme is encoded by a small of gene family, and three copies of *CHS* in *E. sagittatum* were found (Zeng et al., 2013). Therefore, the *EsCHS* member chose for the transient reporter assay may be not the real target of *EsMYBF1* TF, because *AtMYB12* regulator as a positive control still does not strongly induce the promoter of this *EsCHS* (**Figure 5**). It is noticeable that *EsDFRs* and *EsANS* genes encode enzymes responsible for anthocyanin and PAs synthesis, but their promoters were not activated by *EsMYBF1* in the transient reporter assay (**Figure 5**). These results further confirmed that *EsMYBF1* is a flavonol-specific MYB regulator, just like *AtMYB12* and *VvMYBF1* regulators. In summary, *EsMYBF1* specifically activates the flavonol pathway genes without the need of a *bHLH* cofactor.

The function of *MYB* regulating the flavonol synthesis is generally highly conserved among the different plant species. Similar results were obtained for the flavonol-specific *MYB* regulators when overexpressed in a heterologous system, such as *AtMYB12* overexpression in tobacco (Misra et al., 2010) or in tomato (Luo et al., 2008), and *AtMYB11* overexpression in tobacco and tomato (Li et al., 2015b). Ectopic expressions of these *MYB* regulators in transgenic tobacco or tomato can modulate the phenylpropanoid pathway genes and then the flavonoid accumulation, in particular upregulate the flavonol pathway genes and enhance the flavonol accumulation. Corresponding, overexpression of *EsMYBF1* in tobacco resulted in the elevated expression of the flavonol pathway genes and the enhanced accumulation of flavonols (**Figures 6** and **7**). Global gene expression analysis for *AtMYB12* overexpression in tobacco or in tomato indicated that *AtMYB12* can modulate numbers of molecular processes, including aromatic amino acid biosynthesis, phytohormone signaling, stress responses and phenylpropanoid biosynthesis, in addition to the flavonol biosynthetic pathway (Luo et al., 2008; Misra et al., 2010; Pandey et al., 2015a). A previous study showed that the pathway genes leading to flavonol synthesis are upregulated in transgenic tobacco by ectopic expression of *AtMYB12*, including *PAL* (phenylalanine ammonia lyase), *C4H* (cinnamate 4-hydroxylase), *CHS*, *CHI*, *F3H*, and *FLS* genes (Misra et al., 2010). The ectopic expression of *EsMYBF1* in tobacco also produced a similar results (**Figure 7**). Moreover, three genes *NtF3H*, *NtCHI* and *NtFLS* involved in flavonol synthesis were more considerably upregulated than other upregulated genes of flavonoid pathway (**Figure 7**). Similarly, tomato *CHS*, *FLS*, and *F3H* genes were most strongly induced by *AtMYB11* or *AtMYB12* overexpression in transgenic tomato (Li et al., 2015b; Pandey et al., 2015a). These target genes for *EsMYBF1* TF regulation are indispensable for the biosynthesis of flavonols. In addition, due to the direction of the metabolic flux from anthocyanin to flavonol pathway, the accumulation of anthocyanin in transgenic tobacco flowers was remarkably decreased (**Figure 6B**). Similar results were also reported for *AtMYB12* overexpression in tobacco and tomato (Luo et al., 2008; Pandey et al., 2015a), and *AtMYB11* overexpression in tobacco (Pandey et al., 2015b). This reduction of anthocyanin accumulation can be explained by the significant downregulation of *NtDFR* and *NtANS* genes which are required for anthocyanin synthesis. Finally, these results proved that *EsMYBF1* is a functional regulator of flavonol synthesis.

## Conclusion

A *R2R3-MYB* TF, *EsMYBF1* was isolated from *E. sagittatum* and functionally characterized. The present work demonstrates that *EsMYBF1* is a flavonol-specific *MYB* regulator which can control the flavonol pathway genes. Preferential expression in leaves of *EsMYBF1* and its correlation with the accumulation patterns of the main BCs during leaf development suggest that *EsMYBF1* is involved in the developmental regulation of the biosynthesis of the flavonol-derived BCs in *Epimedium* leaves. Additionally, *EsMYBF1* functions as a regulator of flavonol synthesis without the *bHLH* cofactor. The functional characterization of *EsMYBF1* not only provides insight into understanding the regulation of the biosynthesis of the flavonol-derived BCs in *Epimedium* plants, and also provides a potential effective TF for genetic manipulation to improve the accumulation of flavonols in the transgenic plants.

## Materials and Methods

### Plant Materials

Plants of *E. sagittatum* were originally collected from different regions of China and grown in the experimental field of the *Epimedium* repository at Wuhan Botanical Garden, in the central of China. *Nicotiana tabacum* cv. NC89 was used for genetic transformation, and transformed tobacco plants were grown in a greenhouse until required.

### Isolation of the Full-Length cDNA and Genomic Clones of *EsMYBF1*

A survey had been previously investigated that the correlation of gene-to-metabolite during the leaf development stages of *E. sagittatum* was analyzed (Huang et al., 2015). We found that a candidate gene, encoding a *MYB* transcription factor, basically corresponded to the accumulation patterns of the main four BCs (epimedin A, B, C and icariin) and co-expressed with several genes of the flavonol biosynthetic pathway. Therefore, this gene was firstly considered to be a strong candidate gene responsible for the flavonol-derived BC synthesis. In order to obtain the full-length cDNA clone of this gene, both 5′-RACE and 3′-RACE techniques were carried out, following the protocol of SMART RACE cDNA Amplication kit (Clontech, Japan). The expected 5′-RACE and 3′-RACE PCR products were ligated into the pMD19-T vector (Takara, Japan) and sequenced. The assembled sequence was predicted to have an entire ORF, encoding a R2R3-MYB protein. Finally, the full-length cDNA clone of this *MYB* gene was isolated from *E. sagittatum* cDNA template using primers listed in **Supplementary Table S1** and PrimeSTAR HS DNA Polymerase (Takara, Japan). This *MYB* gene of *Epimedium* revealed a high level of similarity with grape *VvMYBF1* regulator, and thus designated as *EsMYBF1*. The genomic DNA sequence of *EsMYBF1* gene was also isolated from genomic DNA of *E. sagittatum* leaves using the same primers for cDNA amplification. The cDNA and genomic sequences of *EsMYBF1* have been deposited in GenBank database with the accession number KU365319 and KU365320, respectively.

### Sequence Alignment and Phylogenetic Analysis

The deduced amino acid sequences of *EsMYBF1* and other selected *MYB* genes were collected from GenBank database, and their accession numbers were indicated in **Figure 1**. Overall protein sequences of these *MYB* genes were used for multiple alignment and phylogenetic analysis. Clustal W program (Thompson et al., 1994) was used to perform the multiple alignment analysis, and BioEdit software (version 7.1) was then used for further edition of aligned sequences. Phylogenetic tree was constructed using the neighbor-joining method through MEGA 5 software (Tamura et al., 2011). Default parameters for Clustal W and MEGA 5 softwares were used, unless otherwise mentioned. In addition, the exon/intron genomic structure of EsMYBF1 gene was analyzed using Spidey tool^1^.

### Quantitative RT-PCR Assay

Quantitative RT-PCR (qRT-PCR) assay was carried out to determine the expression levels of *EsMYBF1* gene in various tissues of *Epimedium* plants and the flavonoid biosynthetic pathway genes in transgenic tobacco. Total RNA was extracted using RNAiso Plus (Takara, Japan) from leaves, flower buds and flowers of *Epimedium*, and from flowers of transgenic tobacco. While RNAiso Plus reagent and Fruit-mate for RNA Purification (Takara, Japan) were used together to extract total RNA from fruits and roots of *Epimedium*. One microgram of total RNA was firstly digested with gDNA eraser (Takara, Japan) to remove any contaminated genomic DNA, and then was reverse transcribed with PrimeScript RT reagent kit (Takara, Japan) following the supplier’s instruction. The reverse transcribed cDNA product was diluted fivefold as the template in qPCR assay. The qPCR assay was set up using SYBR Premix Ex Taq II (Tli RNaseH Plus) kit (Takara, Japan), and run in an ABI7500 Fast Real-Time PCR equipment (Applied Biosystems, USA) following the manufacturer’s recommendation. The primers used for qPCR assay are listed in **Supplementary Tables S1** and **S2**. The melting curve program was included at the end of qPCR program to ensure the specific amplification. The comparative Ct method (2^-ΔΔCT^) was used to determine the relative expression levels of genes (Schmittgen and Livak, 2008). In addition, *Epimedium Actin* gene and tobacco *Tubulin* gene were used as internal controls for qPCR assay in *Epimedium* and tobacco, respectively.

### Yeast Two-Hybrid Assay

In order to confirm that EsMYBF1 is cofactor independent, Y2H assay of EsMYBF1 with two bHLH regulators of the flavonoid pathway was carried out as described previously (Huang et al., 2016). The coding region of EsMYBF1 was subcloned into the pGADT7 and pGBKT7 vectors (Clontech, Japan), generating the EsMYBF1-AD and EsMYBF1-BD constructs, respectively. The primers used for EsMYBF1 construction were listed in **Supplementary Table S1**. The pGADT7 construct containing the GAL4 activation domain fused with AtTT8 or EsTT8 protein, and the pGBKT7 construct harboring the GAL4 DNA-binding domain fused with AtTT8 or EsTT8 protein which had been developed previously (Huang et al., 2016), were used directly in this study. The different combined constructs were co-transformed into yeast strain AH109 using the LiAc/SS carrier DNA/PEG method (Gietz and Schiestl, 2007). Transformants were sequentially screened on the double (SD/-Trp/-Leu), quadruple (SD/-Trp/-Leu/-Ade/-His) dropout medium and quadruple dropout medium plus with X-Gal substrate. The growth of yeast cells was observed 3–4 days after incubation. Transformed yeast cells containing pGBKT7-53 + pGADT7-T, pGBKT7-Lam + pGADT7-T constructs were used as a positive and a negative control, respectively.

### Dual Luciferase Reporter Assay

Transcription activity of *EsMYBF1* TF against promoters of the flavonoid biosynthetic genes was performed using a dual luciferase reporter assay of transient transformed leaves of *N. benthamiana*. The 5′-flanking regions of *EsCHS*, *EsF3H*, *EsFLS*, and *EsDFR2* genes were isolated by Tail-PCR (thermal asymmetric interlaced PCR) or inverse PCR and sequenced. Upstream regions from the ATG start site of these four genes, *EsCHS* (718 bp, Accession number: KC335204), *EsF3H* (1772 bp, Accession number: KU976281), *EsFLS* (624 bp, Accession number: KU976282), and *EsDFR2* (1138 bp, Accession number: KC335206) were amplified from genomic DNA of *E. sagittatum* and subcloned into the transient expression reporter vector pGreenII 0800-LUC which contains the CaMV 35S promoter-REN cassette and the promoterless-LUC cassette (Hellens et al., 2005). Meantime, the coding regions of *EsMYBF1* and *AtMYB12* (Accession number: NM_130314.3) were transferred from pMD19-T vector (Takara, Japan) into the transient expression effector vector pGreenII 62-SK which contains the CaMV 35S promoter-MCS-CaMV terminator cassette (Hellens et al., 2005). All primers used for reporter and effector constructions were listed in **Supplementary Table S1**. In addition, the reporter constructs containing *EsDFR1* or *EsANS* promoters and the effector constructs containing *EsTT8* or *AtTT8* TFs had been developed previously (Huang et al., 2013), and were also directly used for transient expression assay in this study.

All the reporter and effector constructs were transformed into *Agrobacterium tumefaciens* GV3101 by electroporation method. *Agrobacterium* was cultured on LB agar supplemented with selection antibiotics and incubated at 28°C for 2 days. The confluent bacteria was re-suspended in infiltration buffer (10 mM MgCl_2_, 0.5 μM acetosyringone) to an OD_600_ of 0.2–0.3, and incubated at room temperature without shaking for 2–3 h before infiltration. Transient transformation was conducted by mixing 100 μL of *Agrobacterium* culture transformed with the reporter cassette and 450 μL of a second *Agrobacterium* cultures transformed with the effector cassette containing *EsMYBF1*, and *EsTT8* or *AtTT8* TFs. Approximately 300 μL of this *Agrobacterium* mixture was infiltrated into 3–4 young leaves of each plant, with at least two points for each leaf. At least four plants were used for each treatment. The transient expression was assayed 3 days after inoculation.

Dual luciferase assay of transient transformed *N. benthamiana* leaves was carried out according to a previous report (Hellens et al., 2005) using the Dual Luciferase Reporter Assay System (Promega, USA). In brief, 1 cm leaf disks were punched 3 days after inoculation, and ground in 500 μL of Passive Lysis Buffer. 10 μL of a 1/100 dilution of this crude extract was measured in 40 μL of Luciferase Assay Buffer. Another 40 μL of Stop and Glow buffer was then added and a second chemiluminescence measurement was made. Luminescence units were measured using a GloMax 20/20 luminometer (Promega, USA), with a 5 s delay and 10 s integrated measurement. Activity data were expressed as the ratio of LUC to REN activity. Background controls were run with only the promoter-LUC reporter construct (no TF). In some cases, positive controls were run using the *AtMYB12* TF with known activity.

### Overexpression Vector Construct and Tobacco Transformation

For ectopic expression of *EsMYBF1* in tobacco plants, the full-length cDNA of *EsMYBF1* was subcloned from the pMD19-T vector (Takara, Japan) digested with *Sal* I and *Sac* I to the modified binary vector pMV (derived from the pBI121 binary vector) digested with *Xho* I and *Sac* I, generating the pMV-EsMYBF1 overexpression construct. Thus, *EsMYBF1* is expressed under the control of the CaMV 35S promoter. This overexpression construct was introduced into *Agrobacterium tumefaciens* strain EHA105 by electroporation for tobacco transformation. The leaf disk method was used as previously described (Horsch et al., 1985) for genetic transformation of tobacco. Transformed tobacco plants were screened using kanamycin antibiotics as a plant selective marker, and the presence of the introduced *EsMYBF1* in transgenic tobacco was confirmed by PCR assay. Finally, three representative independent T_2_ transgenic lines showing obvious color change of tobacco flowers were used for further analysis. The transgenic tobacco carrying the pMV empty vector was used as the negative control.

### Flavonoid Content Measurement in Transgenic Tobacco Flowers

To evaluate the effects of *EsMYBF1* overexpression on flavonoid content in transgenic tobacco, the flavonoid extraction and content measurement are carried out as previously described (Huang et al., 2015). Firstly, flower samples of transgenic tobacco were collected at the blooming stage, frozen immediately in liquid nitrogen and stored at -70°C until use. Total anthocyanin content was measured using a spectrophotometric method, while flavonol content was determined using a HPLC (High Performance Liquid Chromatography) method. In brief, total anthocyanin was extracted using 1% HCl/methanol in the dark at 4°C overnight with occasional shaking. The extracts were centrifuged at 12,000 *g* for 10 min, and the supernatant was filtered with a 0.22 μm filter membrane and measured at 530 and 657 nm for absorbance determination. The equation A530-0.25 × A657 was used to compensate for the absorption of chlorophyll and its degradation products at 530 nm. Total anthocyanin content was calculated using the subtracted absorbance/fresh weight. Meanwhile, flower powders were immersed into 80% methanol, and sonicated for 30 min and then kept at 4°C overnight to extract flavonols. Flavonol content in tobacco was calculated as aglycones by preparing acid-hydrolyzed extracts. An aliquot of 400 μL of the supernatant was transferred to a fresh tube, acid-hydrolyzed by adding 120 μL of 3 N HCl, incubated at 90°C for 1 h, and then mixed with 200 μL of methanol. This hydrolyzation solution was filtered through a 0.22 μm filter membrane prior to injection. An Agilent 1100 series HPLC system equipped with an Agilent TC-C18 column (5 μm, 4.6 mm × 250 mm) was used for chromatographic analysis. The mobile phase of HPLC system consisted of solvent A (0.1% formic acid in water), solvent B (acetonitrile) and solvent C (methanol). The gradient elution program was: 0 min, 10% B + 2% C; 10 min, 20% B + 4% C; 15 min, 50% B + 10% C; 20 min, 20% B + 4% C; 25 min, 10% B + 2% C; 28 min, 10% B + 2% C. The column was maintained at 25°C and the solvent flow rate was 1.0 mL/min, and the injection volume was 10 μL. The detection wavelength was set at 350 nm for kaempferol and quercetin flavonols. Flavonol compounds were identified with reference to commercial standards of kaempferol and quercetin, and their quantification was measured according to the standard curve of each reference. The flavonoid analysis of each sample was repeated three times using five independent biological replicates.

## Author Contributions

WH and YW initiated and designed the research. WH, AK, JC performed and analyzed the experiments. CZ and HL contributed to reagents, materials and analysis tools. WH wrote the paper, LY and YW revised the paper.

## Conflict of Interest Statement

The authors declare that the research was conducted in the absence of any commercial or financial relationships that could be construed as a potential conflict of interest.

## Footnotes

1 http://www.ncbi.nlm.nih.gov/spidey/
