# Supplementary material for: A R2R3-MYB Transcription Factor Regulates the Flavonol Biosynthetic Pathway in a Traditional Chinese Medicinal Plant, Epimedium sagittatum
Source: Front Plant Sci. 2016 Jul 21;7:1089. doi: 10.3389/fpls.2016.01089 (PMC4954812; doi:10.3389/fpls.2016.01089)
Supplement: Supplementary file 1 [file Table_1.DOCX]

**Table S1: Primers used for isolation and characterization of EsMYBF1 genes**

| **Primer names** | **Forward sequence (5’-3’)** | **Reverse sequence (5’-3’)** |
| --- | --- | --- |
| Full-length EsMYBF1 cDNA cloning | CGCCCTTCAAGCTTTTCTGGT | TTACAACATTCTTCTTATGTAACATTCG |
| EsMYBF1 primers for qPCR assay | AATAGGTGGTCACTGATTGCTGC | CACCCATCTTGGCTAAGTTCATC |
| EsActin primers for qPCR assay | GCCATTCAGGCTGTTCTTTC | GGTAAGATCGCGACCTGCTA |
| EsMYBF1 ORF for yeast two-hybrid (AD) | GCCATATGGGAAGATCGCCATGTTG | GGCTCGAGACATTGAAACTCTTCCAACCAAG |
| EsMYBF1 ORF for yeast two-hybrid (BD) | GCCATATGGGAAGATCGCCATGTTG | GGGTCGACACATTGAAACTCTTCCAACCAAG |
| EsCHS promoter for transient reporter assay | ACATGTGTGGATTTGGCTTAACG | TTAGCTCTTACTGTTATTATTTATCACG |
| EsF3H promoter for transient reporter assay | CTCCGCAATCTCCATACATTCGTC | TGCGGGTTAATAGTTTGTTTCCT |
| EsFLS promoter for transient reporter assay | GTAGGTTTTGAGACTCACAGTAGGTGC | GAAACTTTGGTGTTTTCTTCTTCTTCTC |
| EsDFR2 promoter for transient reporter assay | ATCTCAAAATTACCTTTCGTTGCTA | TTCTTAAGGATGGTGTTAATTGTGAC |
| AtMYB12 ORF for transient reporter assay | ATGGGAAGAGCGCCATGTTGC | TCATGACAGAAGCCAAGCGACCAA |

Underlines represent enzyme recognition site

**Table S2: Primers used for qPCR assay of flavonoid-related genes in transgenic tobacco**

| Gene name | Accession No. | Forward primer (5’-3’) | Reverse primer (5’-3’) |
| --- | --- | --- | --- |
| *NtCHS* | AF311783 | AGCGAGCATAAGGTTGAG | ACCACCACTATGTCTTGTC |
| *NtCHI* | AB213651 | CTTTTCTCGCCGCTAAATG | TTTCTGCCACCTTCTCTG |
| *NtF3H* | AB289450 | GAGGCAATGGGCTTAGAG | TCAGTGTGTCGTTTCAGTC |
| *NtF3'H* | AB289449 | AGCCATAGTCAAGGAAACC | CTCACAACTCTCGGATGC |
| *NtFLS* | DQ435530 | GTCCCATATAACCATTCTTGTC | CACTCTTGTATTTCCCATTGC |
| *NtDFR* | EF421429 | TAAGAAGATGACAGGATGGATG | TGGCGGTATGATGCTAATG |
| *NtANS* | AB289447 | CTACATTCCAGCAACAAGTG | GTCCCAGCCCAATAGAAAG |
| *NtTub1* | AJ421411 | TCCGTGGTGATGTTGTG | TGGTGGCTGATAGTTGATAC |
